# The Lung Immune Response to Nontypeable *Haemophilus influenzae* (Lung Immunity to NTHi)

**DOI:** 10.1155/2015/706376

**Published:** 2015-05-31

**Authors:** Paul T. King, Roleen Sharma

**Affiliations:** ^1^Monash Lung and Sleep, Monash Medical Centre, Melbourne, VIC 3168, Australia; ^2^Monash University Department of Medicine, Monash Medical Centre, Melbourne, VIC 3168, Australia

## Abstract

*Haemophilus influenzae* is divided into typeable or nontypeable strains based on the presence or absence of a polysaccharide capsule. The typeable strains (such as type b) are an important cause of systemic infection, whilst the nontypeable strains (designated as NTHi) are predominantly respiratory mucosal pathogens. NTHi is present as part of the normal microbiome in the nasopharynx, from where it may spread down to the lower respiratory tract. In this context it is no longer a commensal and becomes an important respiratory pathogen associated with a range of common conditions including bronchitis, bronchiectasis, pneumonia, and particularly chronic obstructive pulmonary disease. NTHi induces a strong inflammatory response in the respiratory tract with activation of immune responses, which often fail to clear the bacteria from the lung. This results in recurrent/persistent infection and chronic inflammation with consequent lung pathology. This review will summarise the current literature about the lung immune response to nontypeable *Haemophilus influenzae*, a topic that has important implications for patient management.

## 1. Introduction

Nontypeable* Haemophilus influenzae* (NTHi) is a bacterium that is present in the nasopharynx of most healthy adults and in this situation it appears to be a commensal [1]. It may also spread down to the lower respiratory tract and in these locations it has been well recognized to be associated with inflammation and disease.

NTHi has had a relatively low profile amongst clinicians with a respiratory/pulmonary background and for many years was considered not to be an important pathogenic bacterium [2]. Recently, there has been increasing evidence to show that this bacterium is highly prevalent and pathogenic in a variety of important lower respiratory conditions including chronic obstructive pulmonary disease (COPD), bronchiectasis, cystic fibrosis, and pneumonia.

It is not clearly understood why NTHi appears to be a commensal in the pharynx but in the lower respiratory tract is an important respiratory mucosal pathogen. The bacteriology of NTHi has been intensively studied over a number of years. Although there have been numerous studies investigating the immune response to NTHi, no singular model for an effective immune response or lack of response has been developed. The host immune response is likely to be a critical factor in preventing NTHi from causing and/or contributing to clinical disease. The presence of NTHi in the lower respiratory tract induces activation of innate and adaptive immune responses that often fail to clear the bacteria from the lung. This results in recurrent/persistent infection and chronic inflammation with consequent lung pathology. Lung host immunity to NTHi is only partially understood and this review will summarize the current literature. Upper respiratory tract disease with this bacterium is beyond the scope of this review.

## 2. Bacteriology

*Haemophilus influenzae* is a Gram-negative coccobacillus that is fastidious and requires X-factor (haemin) and V-factor (nicotinamide adenine dinucleotide) for growth.* H. influenzae* can be divided into typeable and nontypeable strains. The typeable strains are defined by the presence of a polysaccharide capsule, with six subtypes (a–f, based on their ability to react with antisera of defined polysaccharide capsules) of which type b is best known [3, 4]. The typeable strains typically cause systemic disease such as meningitis. In contrast, the nontypeable strains lack a capsule and are predominantly mucosal pathogens. There is enormous diversity in NTHi strains principally through variability in outer membrane proteins [5].

*Haemophilus influenzae* colonizes the nasopharynx early in life and there is significant turnover of different strains particularly in young children [6]. Children may be colonized with multiple different strains simultaneously [7]. As such in this situation, NTHi can be considered to be a part of the normal microbiome, present in the upper airway. The role of this bacterium in the upper airway microbiome is not well understood. The density of colonization of the upper respiratory tract and the number of different isolates is correlated with middle ear infections [8]. This bacterium has a number of mechanisms which it uses to facilitate its survival in the human host and there is extensive published literature about this topic (which is beyond the scope of this review). The presence of* Haemophilus influenzae* as a commensal in the nasopharynx serves as an ongoing source of potential infection for the lower respiratory tract.

## 3. Lower Respiratory Tract Disease and NTHi

The great majority of respiratory infections arise from the nontypeable forms of* H. influenzae* [1, 9]. Infection with NTHi is often recurrent and chronic. There is a broad range in prevalence between different studies reflecting the difficulties in accurately isolating this pathogen. As NTHi is an opportunistic pathogen it often infects lungs, which have structural damage such as COPD (“noninfectious lung disease”) as well as primary infectious conditions such as bronchiectasis [10]. The recent use of bacterial sequencing, particularly the use of 16S rRNA, has also highlighted the prevalence of* H. influenzae* in the lung [11, 12].

An underappreciated property of NTHi is its ability to invade into lung tissue and intracellular survival [13]. NTHi has been shown to be present between epithelial cells and inside macrophage-like cells in patients with chronic bronchitis and adenoid formation [14–16]. In lung explants from a variety of conditions, Möller et al. demonstrated extensive invasion of NTHi into the lung parenchyma [17]. Another recent study demonstrated in all stages of COPD invasion of lung tissue with NTHi [18]. NTHi is a facultative anaerobe and this property may be potentially important in tissue infection [13].

### 3.1. Chronic Obstructive Pulmonary Disease

The role of bacterial infection in COPD has been controversial. The “British Hypothesis” proposed that bacterial infection had a primary role in the pathogenesis of COPD [19], whilst subsequently the work of Fletcher and Peto found no association [20] and for many years bacteria were regarded as being peripheral to the pathogenesis of COPD by clinicians with a respiratory/pulmonary background despite publications highlighting its potential importance [21, 22]. Recently, there has been renewed interest in the role of bacteria in the pathogenesis of COPD.

The most common association is with chronic bronchitis in patients with COPD. This is in the context of both acute exacerbations and chronic colonization. NTHi is the most common bacterium isolated in exacerbations of COPD, with 25–80% of exacerbations with* H. influenzae* isolation. This bacterium is also the most common bacteria colonizing the airways in COPD [23–26]. The bacterial load/colonization with NTHi is correlated with the severity of (1) airway inflammation, (2) exacerbations [27], and (3) daily symptoms [28]. Bacterial colonization increases daily symptoms [28]. Exacerbations of COPD are also associated with the presence of bacteria inside epithelial cells [23].

### 3.2. Bronchiectasis and Cystic Fibrosis

**Figure 1:**
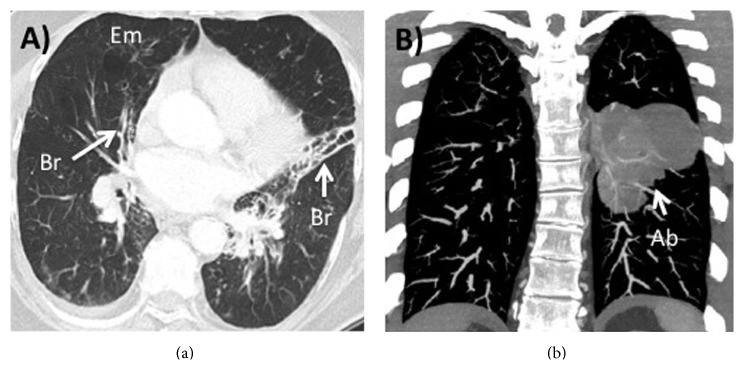
Clinical situations with NTHi infection. (a) shows a computed tomography (CT) scan of a subject with severe chronic obstructive pulmonary disease and the prescence of bronchiectasis (Br) and emphysema (Em). This patient had chronic NTHi airway colonization and exacerbations for a number of years. (b) shows a CT scan of a patient with Hyper IgE syndrome with a lung abscess (Ab) from which* H. influenzae* was cultured.

NTHi is also the dominant bacterium isolated in patients with bronchiectasis [29]. There is some overlap between COPD and bronchiectasis; half of patients with COPD may have coexistent bronchiectasis and the presence of bronchiectasis is associated with worse outcomes in COPD. In patients with cystic fibrosis (CF), this bacterium is less prevalent than in non-CF bronchiectasis but is important in the early stages of the disease. However, Moller et al. showed that NTHi was also present in end-stage lung explants in patients with CF [17]. A CT scan of a patient with bronchiectasis and COPD is shown in Figure 1(a).

*Pneumonia*. NTHi is an important cause of pneumonia in adults, particularly in patients with chronic lung disease. It is the third most common cause of pneumonia. Typically, patients have bronchopneumonia pattern rather than lobar pneumonia. COPD and bronchiectasis/CF patients have a markedly increased risk of pneumonia.

### 3.3. Interstitial Lung Disease

A recent study has described that lung bacteria including* H. influenzae* are associated with decline in lung function and death in idiopathic pulmonary fibrosis [30].

## 4. NTHi and the Activation of Intracellular Signaling Pathways

NTHi is a bacterium that when present in the nasopharynx appears to be a commensal. However, when it moves into the lower respiratory tract, it can then elicit strong inflammatory responses such as what occurs in COPD. In this circumstance it can cause prolonged inflammation [31]. Singh et al. studied the relationship between three common pathogenic bacteria (*H. influenzae, S. pneumoniae*, and* M. catarrhalis*) and inflammation in stable patients with COPD [32]. They found that* H. influenzae* was associated with significantly higher levels of airway inflammation (as measured by levels of interleukin (IL)-1*β*, myeloperoxidase, and CXC-chemokine ligand 8) for all given pathogen loads and was significantly higher than with the other two bacteria. There was not a significant relationship with systemic inflammation as measured by C-reactive protein and plasma fibrinogen.

Surface receptors on innate (e.g., toll-like receptors (TLRs)) and adaptive (e.g., lymphocyte receptors) cells may be activated by stimuli such as pathogenic microorganisms to drive the production of a variety of intracellular transcription factors. This results in the cellular production of inflammatory mediators such as cytokines, chemokines, and reactive oxygen species which mediate both innate and adaptive immunity.

The intracellular signaling factor most strongly associated with NTHi infection is NF-*κ*B. Moghaddam et al. have administered aerosolized lysate NTHi (defined strain that had been exposed to ultraviolet light then sonicated) to mice on a weekly basis for 8 weeks. This exposure induced rapid activation of NF-*κ*B in airway cells and increases of inflammatory cytokines and neutrophils in bronchoalveolar lavage (BAL) fluid. Repetitive exposure induced infiltration of macrophages, CD8+ T cells, and B cells around airways and blood vessels and collagen deposition in airway and alveolar walls. The lysate NTHi thus induced features of COPD with peribronchial and perivascular inflammation and lymphoid aggregates with fibrosis [33]. NTHi acts primarily through the NF-*κ*B signaling pathway to increase inflammation [34]. This effect may be enhanced by the effect of cosecreted cytokines from epithelial cells, such as by the addition of TNF-*α* [34]. NTHi may stimulate epithelial cells to produce cytokines such as TNF-*α* and IL-1*α*, which drives a positive feedback loop further enhancing inflammation [35]. Macrophages are also a major source of TNF-*α* and IL-1*α* production.

Growth factors also play an important role in driving inflammation in combination with NTHi. Transforming growth factor-beta (TGF-*β*) activates the NF-*κ*B pathway with NTHi in epithelial cells and lung tissue [36, 37]; and this is an unusual effect as TGF-*β* is most commonly an inhibitor of inflammation [38]. The epidermal growth factor receptor (EGFR) pathway also has proinflammatory effect and TLR2 has a crucial role in this process [39].

Other recent studies have assessed inflammatory mechanisms in response to NTHi. A study of human alveolar macrophages, cell-line macrophages, and human lung tissue demonstrated upregulation of the NLRP3 inflammasome with Caspase-1 dependent secretion of IL1-*β* [40]. Heyl et al. demonstrated that a c-lectin receptor Dectin-1 was expressed in the human lung and was correlated with a proinflammatory response to NTHi [41].

Clinical infections with NTHi often occur in association with other bacterial and viral pathogens. This coinfection has a synergistic effect to enhance inflammation. Coinfection of NTHi with* Streptococcus pneumoniae* and* Moxarella catarrhalis* results in NF-*κ*B dependant pathway upregulation of TNF-*α*, IL-1*β*, and IL-8 [42, 43] and also through TLR-2 [44].

The immune/inflammatory response to infection is tightly controlled to prevent damage to the host. Important controlling pathways include deubiquitinases such as CYLD, which is the best characterized in NTHi respiratory infections [45, 46]. CYLD downregulates NF-*κ*B and its primary action is by the removal of ubiquitin (ubiquitin is a regulatory protein with widespread distribution in eukaryotic cells) [47]. In CYLD knockout mice, NTHi infection enhances leukocyte infiltration and inflammation [46]. Another deubiquitinase that is important in controlling the inflammatory response to NTHi is A20, which may downregulate NF-*κ*B through a TNF-*α* pathway [48].

A variety of cell types including epithelial cells, macrophages, and lymphocytes are exposed to NTHi both in the nasopharynx and the lower respiratory tract. All these cell types can produce inflammatory mediators but why NTHi appears to be a commensal in the nasopharynx but a potential strong inducer of inflammation in the lung is not understood. Further defining the intracellular signaling pathways in response to chronic NTHi infection may give critical insights as to why this occurs. This also has therapeutic implications, as there are a number of new therapies becoming available which target inflammatory signaling pathways [49].

## 5. Innate Immunity to NTHi

The innate immune response is involved in the first-line protection against infection and has both structural and cellular components.

### 5.1. Structural Airway Defence

A key component of innate immunity is the structural airway defence. Patients with cystic fibrosis have deficiency of mucociliary function and in early-stage disease NTHi is a prominent airway pathogen. The specific mechanisms that increase the risk of NTHi infection in CF have not been well defined. This is potentially an important area of research. Primary ciliary dyskinesia (PCD) is an inherited condition due to a deficiency in the dynein arm of cilia. It results in chronic suppurative lung disease particularly bronchiectasis. A study of 20 patients with PCD found that the dominant pathogen was* H. influenzae* present in 80% of subjects [50]. Therefore, deficiency of mucociliary function is strongly associated with* H. influenzae* infection.

NTHi also produces factors which damage/inhibit ciliary function and these include LOS and protein D [51, 52]. NTHi may also cause direct damage to ciliated epithelium. A more recent study has demonstrated that NTHi decreases cilia beating via protein kinase C*ε* and this effect was only observed with fresh bacterial culture [53].

The airway epithelium is important in defence against potential pathogens particularly in the case of potentially invasive bacteria such as NTHi. NTHi has a number of strategies to facilitate its binding/adherence to epithelial cells and there is emerging evidence that NTHi may persist in the human respiratory tract by surviving inside epithelial cells [13]. NTHi induces the upregulation of NF-*κ*B signaling pathways by human bronchial epithelial cells [34]. Goyal et al. have described that NTHi induces apoptosis of Type II alveolar cells [54]. NTHi infection has also been shown to inhibit epithelial host defence proteins. Airway epithelial cells respond to NTHi by the secretion of inflammatory mediators including IL-6, IL-8, and TNF-*α* [55, 56]. NTHi activates toll-like receptors (TLRs) on epithelial cells that enhance the production of inflammatory mediators. The role of the epithelium in host defence to NTHi is generally not well characterized.

### 5.2. The Complement System

The three main functional mechanisms of complement are (1) activation of inflammation, (2) opsonisation of pathogens for phagocytosis, and (3) lysis of susceptible pathogens by the formation of the membrane attack complex (MAC). Complement can be activated by three different pathways. The classical pathway (CP) of complement activation is mediated by antigen-bound IgM or IgG binding to and activating the C1 complex. C-reactive protein (CRP) may also activate the CP [57]. The lectin pathway is activated by the binding of mannose-binding lectin (MBL) or ficolins to carbohydrates on the surface of microbial pathogens. The alternative pathway is activated by the surface components of the microbial pathogens.

Nontypeable strains are susceptible to serum killing and this effect appears to be primarily due to the formation of the MAC with lysis of the bacteria. There is variability in this effect between different strains with some strains being resistant. The sensitivity of NTHi to complement-mediated killing may be a reason why this bacterium is a relatively rare cause of systemic infection. There have been a number of publications on* H. influenzae* and complement, and this topic has been reviewed by Hallström and Riesbeck [58]. Whilst complement has predominantly been studied in the context of systemic immunity, there are several studies describing that acute airway inflammation is associated with the presence of complement components in the airway lumen [57, 59].

Evasion of complement is an important survival mechanism and this has been demonstrated to be a feature of a large variety of different pathogens. NTHi has developed two main strategies [58] to mediate protection against complement-mediated attack: (1) barriers that prevent the activation/deposition of complement components and (2) production of inhibitory mediators to prevent complement activation. Mechanisms of resistance to deposition of complement components/MAC formation include alterations in lipoligosaccharide, phosphorylcholine, sialic acid, and other outer membrane proteins such as P2, P5, and P6 [60, 61]. NTHi produces mediators to inhibit complement including enhancing the effect of C4BP, Factor H, and vitronectin [62].

Deficiencies of complement are associated with increased risk of infection, most commonly with bacteria that are encapsulated [63]. Patients with deficiencies of the early components of the CP (C1, C4, and C2) primarily have increased infections with* Streptococcus pneumoniae*,* H. influenzae, Neisseria meningitides*, and* S. agalactiae*. Deficiencies of the alternative pathway and C3 are associated with* N. meningitides* and* S. pneumoniae*, whilst deficiencies of the terminal attack components are strongly associated with* N. meningitides.* Generally, in the studies, typing of the* H. influenzae* was not performed, so the proportion of typeable versus nontypeable strains is not known. MBL deficiency is a very common immunodeficiency, which generally has minimal clinical manifestations but may be associated with recurrent severe infections with* S. pneumoniae*. A recent study has highlighted the potential role of MBL deficiency in bronchiectasis [64]. Whether MBL deficiency increases respiratory infection through deficient complement activation remains is to be determined. In contrast to isolates from the upper respiratory tract, lower respiratory tract isolates of NTHi (particularly in the context of exacerbations of COPD) have increased resistance to the complement- and antibody-dependent bactericidal effects of serum [61].

The effect of complement in mediating killing of airway bacteria and further defining the role of MBL on complement are of potential interest. This is an important area to study and may represent a new therapeutic target.

### 5.3. Cellular Immune Response

The cellular immune response to NTHi is predominantly mediated by the alveolar macrophages as the first-line cell and in chronic inflammation, whilst the neutrophil primarily functions in acute exacerbations. The main functions that have been studied are TLR expression, macrophage phagocytosis, and cytokine production.

The binding of TLRs by NTHi has a primary role in driving effector cellular responses. A number of studies have assessed the effect of TLR function in response to NTHi with an emphasis on TLR4 and TLR2. The TLR-4 pathway is activated in response to LPS in the wall of Gram-negative bacteria (or in the case of NTHi by LOS). This results in two intracellular signaling pathways: (1) MyD88 and (2) Toll/IL-1R domain-containing adaptor-inducing IFN-*β* (TRIF). CD14/TLR-4 knockout (KO) mice infected with NTHi have decreased production of TNF-*α*, IL-1*β*, and IL-6 by immune cells compared to control; these KO mice also had impaired bacterial clearance [65, 66]. These investigators also showed that the primary activating pathway for this response was through MyD88. The TLR2 pathway is also important in response to bacteria. NTHi binds to TLR2 to activate NF-*κ*B translocation-dependent (with activation of NF-*κ*B inducing kinase-IKK*α*/*β* complex) and translocation independent pathways (with activation of MKK3/6-MAPK) [67]. TLR2 activation also induces COX-2 and PGE2 expression via p38 MAPK and NF-*κ*B [68]. A recent study has described that subjects with COPD have impaired TLR2 and TLR4 responses to NTHi [69]. The connection between TLR activation and effector immune responses is an area of potential interest.

**Figure 2:**
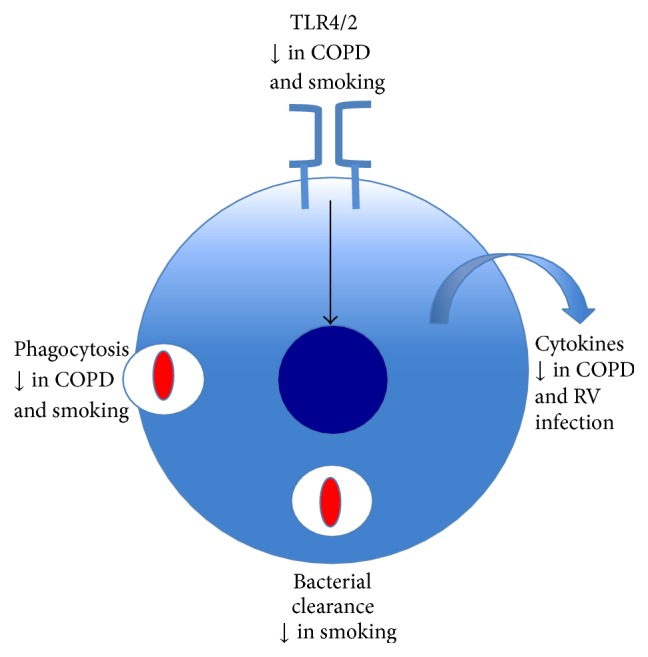
Factors associated with impaired macrophage immune responses to NTHi. There are some important factors that inhibit the lung macrophage response to NTHi infection. Macrophage activation through TLR4 and 2 is impaired in patients with chronic obstructive pulmonary disease (COPD) and by smoking. Phagocytosis is reduced in patients with COPD; smoking also inhibits this process as well as bacterial clearance. Macrophage effector function with cytokine production is impaired in subjects with COPD and by rhinovirus (RV) coinfection.

There are a number of studies that have assessed macrophage function in response to NTHi, with an emphasis on defective phagocytosis. Berenson et al. have shown that alveolar macrophages from COPD had impaired phagocytosis of radioactive labelled NTHi when compared to controls; in contrast there was no difference in results between groups in blood-derived macrophages [70]. Another study, which used a different method of fluorescently-labelled bacteria, found that macrophages from both the lung and peripheral blood of subjects with COPD had impaired phagocytosis of NTHi and* S. pneumoniae* [71]. There are a number of different ways that a bacterium can enter a phagocyte and the mechanisms of the impairment of phagocytosis in COPD noted in these two studies remain to be defined. A follow-up study has described that activation of nuclear erythroid-related factor 2 improves phagocytosis of NTHi by alveolar macrophages [72]. Berenson et al. have described that impairment of phagocytosis of NTHi and* Moxarella catarrhalis* is related to the severity of disease in COPD and is complement independent [73]. The macrophage has a number of strategies to kill phagocytosed pathogens and these include the production of reactive oxygen and nitrogen species, myeloperoxidase, the formation of the phagolysosome, and antimicrobial peptides such as defensins. There is a lack of literature about these important mechanisms in reference to NTHi infection, which have implications for both ability to clear bacteria and excessive production, contributing to local tissue damage. We have recently described that NTHi induces widespread ROS production by a variety of lung cells particularly macrophages and this increases over time with extracellular expression and this may be an important factor in the development of lung oxidative stress [74]. In addition, macrophages also produce mediators such as cytokines as part of their effector function; one study has described that there is impaired human alveolar macrophage function to NTHi antigens with decreased production of IL-8, TNF-*α*, and IL-1*β* [75]. Some key features of macrophage function in relation to NTHi infection are highlighted in Figure 2.

Neutrophils have a predominant role in acute exacerbations of airways disease. Moghaddam et al. in an animal model of NTHi infection have demonstrated prominent lung infiltration of the lung with neutrophils [33]. Neutrophils have generally fairly similar function throughout the body and therefore studies in peripheral blood are very relevant to the lung. A relatively recently described feature of neutrophils is the formation of neutrophil extracellular traps (NETs) in which extracellular DNA in association with proteases is expressed from the cell after phagocytosis of a microbial pathogen, and these NETs are important in the killing of extracellular pathogens. Juneau et al. have described that NTHi induced the formation of NETs and surprisingly these were ineffective in killing extracellular bacteria [76]. We have also recently described that macrophages also make extracellular traps (METs) in response to NTHi infection [74]. These METs express the protease matrix metalloproteinase- (MMP-) 12 and this could potentially have a role in protease imbalance and local tissue damage (e.g., by contributing to the development of bronchiectasis and emphysema). The production of the METs was driven by macrophage ROS production.

There are other innate immune cells in the lung such as the dendritic cell, NK cells, and innate-type lymphocytes, but there is a lack of literature about them. NK cells also respond to NTHi with pronounced proliferation, production of cytokines such as interferon gamma, and the release of cytotoxic granules [77]. The dendritic cells have potentially a very important role as they link the innate and the adaptive immune responses and may be a key cell type involved in the nonclearing immune response that may occur in NTHi infection.

There are a number of well-recognized deficiencies of phagocytes and these include decreased production (e.g., neutropenia), leukocyte adhesion deficiency, chronic granulomatous disease, myeloperoxidase deficiency, Chediak-Higashi syndrome, and the Hyper-IgE syndrome [78]. The only one of these that has been clearly associated with* H. influenzae* infection is the Hyper-IgE syndrome, which arises from a mutation of STAT3 and results in extremely high levels of IgE with recurrent pneumonia/abscesses of the lung and eczema/eosinophilia. Defining how Hyper-IgE syndrome leads to clinical infection is likely to give important insights into the key protective innate immune responses. A CT scan of a patient with Hyper IgE syndrome is shown in Figure 1(b).

## 6. Adaptive Immunity to NTHi

As NTHi is frequently a cause of recurrent/chronic respiratory infection, the adaptive immune response has a key role in determining outcome. The interpretation of adaptive immunity is complicated by the presence of NTHi as a commensal in the upper respiratory tract and most adults have detectable specific antibody to this pathogen [79, 80].

*Key Features of Adaptive Immunity in Response to NTHi*

*Humoral Immunity*

 Antibody is induced in response to a wide variety of NTHi antigens.
 Humoral immunity has role in preventing systemic infection.
 Complement-mediated killing of extracellular bacteria is facilitated by presence of specific antibody.
 “Strain hypothesis”: new strains of NTHi may be associated with exacerbations and generation of new antibody responses with protective immunity.

*Cell-Mediated Immunity*

 Lymphocyte proliferation is important in preventing COPD exacerbations.
 Th1/Tc1 responses in peripheral blood are associated with protective immunity.
 Th1 responses are important in inducing TLR4 expression.
 In lung tissue, patients with COPD have increased production of IL-13, IL-17, and TNF-*α*.

### 6.1. Humoral Immunity

The outer membrane proteins particularly P2, P5, and P6 are important antigens in generating the humoral response [81–84]. There are a number of other components of NTHi that have been demonstrated to induce humoral immune responses, including protein D, protein F, A1 proteases, and the adhesins. Most of these components have significant variability between strains and therefore the immune response is complex. Both healthy controls and patients with lung disease and NTHi infection are able to make systemic humoral immune responses to NTHi [79, 85]. Specific antibody activates the terminal attack complex of complement to cause death of NTHi [85]. A recent study by Otczyk et al. detected IgE to colonizing NTHi in subjects with chronic bronchitis and COPD, suggesting a possible mechanism of bronchospasm induced by this bacterium [86].

Sethi et al. have demonstrated that a proportion of COPD exacerbations are associated with the acquisition of a new strain of NTHi [87]. This then led to the generation of new antibody response to the strain, which resulted in a clearing immunity to this new strain. This effect has been called “the strain hypothesis” [88]. The authors further refined their studies to exclude complicating isolates of* H. haemolyticus *[89]. The same group has also described the repeated isolation of identical strains of NTHi in patients with COPD over a number of years [90]. The ability of NTHi to invade into tissue may be a potential way for this bacterium to avoid the effect of humoral immunity.

There is a lack of literature about localized lung humoral immunity to NTHi particularly the role of secretory IgA. Musher et al. have described that secretory IgA taken from the bronchopulmonary secretions of patients with NTHi induced pneumonia inhibited the opsonizing and bactericidal effects of normal human serum [91]. The results of this study are surprising and the study of lung secretory IgA responses to NTHi is an area of potential interest. Clinically stable COPD patients colonized by* H. influenzae* had lower levels of specific IgA against the microorganism than noncolonized patients [92]. A possible explanation for this finding is that a clonal group of NTHi with two proteases was found to be adapted to infection in COPD [93]. NTHi expresses one and often two distinct IgA proteases to inhibit the effect of IgA. Clementi et al. have recently demonstrated that NTHI IgA1 proteases have distinct roles both in invasion and intracellular persistence of NTHI in respiratory epithelial cells [94]. However, IgA deficiency has not been recognized as a risk factor for clinical* H. influenzae* infection.

Hypogammaglobulinemia with IgG deficiency is the immune deficiency most clearly associated with NTHi infection [95–97]. The administration of IgG replacement therapy has been shown to reduce systemic NTHi infections including a study which showed a reduction in nasopharyngeal colonization (with a possible effect from antibiotics as well) [98].

### 6.2. T Cell Immunity

Abe et al. described that a lymphocyte proliferative response to P6 of NTHi was associated with relative protection from exacerbations of COPD, suggesting that this cell has an important role in host defence [99]. A subsequent study of peripheral blood responses demonstrated that patients with bronchiectasis and chronic NTHi infection had a Th2 predominant response when compared to control subjects who had a Th1 predominant response, suggesting that the Th2 response was associated with nonclearing immunity [100]. A follow-up study by the same group demonstrated similar findings in cytotoxic T cell (Tc) responses with bronchiectasis subjects have a Tc2 type response in peripheral blood [77]. The addition of Th1 mediators was associated with enhanced macrophage killing of NTHi [101]. Knobloch et al. also demonstrated that Th1 responses to NTHi were impaired in COPD by interfering with MyD88/IRAK signaling thereby reducing LPS-induced TLR4 expression [102]. Regulatory T (T reg) cells have a key role in controlling the immune response. A recent study by Kalathil et al. showed that in patients with COPD there were increased numbers of Treg cells in peripheral blood and this was associated with decreased proliferation by T effector cells to NTHi and higher levels of programmed cell death expression (PD-1) [103]. Pizzutto et al. described that children with chronic suppurative lung disease have impaired Th1 responses to NTHi [104]. There are difficulties in obtaining sufficient numbers of cells by bronchoscopy to study low frequency T cell responses, such as antigen-specific immunity and T reg function. A study of the response to live NTHi in surgical lung tissue from patients with COPD compared to controls found that subjects with COPD had strong T cell cytokine responses to this bacterium with the highest levels being of TNF-*α*, IL-13, and IL-17. Interestingly, similar to peripheral blood responses, we found that in COPD the T cell response was Th2/Tc2 predominant [105]. Essifie et al. in a mouse model described that NTHi infection drives IL-17-mediated neutrophilic allergic airways disease [106, 107].

There are some factors which complicate the interpretation of T cell responses. An issue with measuring T cell responses is whether the detected response is a primary immune defect resulting in disease or is an adaption to nonclearing infection. Wynn has hypothesized that the production of IL-13 may be a default position in patients with chronic inflammatory liver disease and infection, that is, potentially less harmful than a Th1 response [108]. In humans the T cell responses are less obviously polarized than in mouse models and as a consequence immune responses to inflammation are often not clearly Th1 or Th2. The literature about Th polarization in COPD emphasizes Th1/Tc1 predominance but some studies have described Th2 effects; part of this variability may be due to the use of different antigenic stimuli between studies.

HIV is associated with increased risk of pneumonia, bronchiectasis, and acute lower respiratory tract infections particularly in children, and the main bacteria involved are* H. influenzae* and* S. pneumoniae* [109, 110], demonstrating the importance of T cells in the prevention of* H. influenzae* lung infection.

## 7. Effects of Smoking and Viral Infection on the Immune Response to NTHi 

Two important clinical situations are associated with NTHi infection and these will be reviewed in this section. The most prevalent factor associated with NTHi infection is smoking-related COPD. In addition viral-bacterial coinfection has recently been recognized to be important.

### 7.1. Effect of Smoking

Smoking causes long-term/permanent changes to lung structure and cellular function, which increases the risk of secondary bacterial infection [111]. Important specific effects include inhibition of macrophage bactericidal function, degradation of extracellular matrix, and expansion of oligoclonal CD8+ T cells and Th17 cells. As NTHi is the most common bacterium isolated in patients with COPD, these changes are likely to be relevant to this bacterium. Smoking has been found to be associated with increased lung inflammation following challenge with NTHi in smoke exposed mice [112]. Martí-Lliteras et al. showed that clearance of NTHi by alveolar macrophages (cell lines and from COPD subjects) is impaired by smoking [113]. Further mechanistic studies have demonstrated that the IL-1 receptor regulates microRNA-135b expression in a negative feedback mechanism in a smoking mouse model [114] and in a chronic smoking exposure model. Smoking inhibited TLR activation of macrophages from patients with COPD with decreased production of TNF-*α*, IL-6, and IL-10 [115]. Lugade et al. have developed a mouse model to examine the effect of chronic smoke exposure and chronic NTHi infection [116]. In this model they showed that cigarette smoking inhibited adaptive immune responses to NTHi with inhibition of IFN-*γ* and IL-4 and specific antibody production but increased levels of IL-17. The establishment of a chronic model of smoking/NTHi is important as it replicates the case of human disease.

Smoking-related COPD is the dominant lung condition associated with NTHi infection and this has the most direct relevance to understanding mechanisms of disease. The study of specific mechanisms arising from chronic smoke exposure in human and animal models is a priority for understanding the immune response to NTHi.

### 7.2. Viral Infection

It has been long known that viral infections may be complicated by secondary bacterial infection. More recent studies have focused on the role of viral and bacterial coinfection in COPD. This coinfection is associated with worse exacerbations of respiratory disease and more inflammation [117]. The most common coinfection is with rhinovirus (RV) and NTHi in COPD [118, 119]. Mallia et al. infected a cohort of patients with COPD with RV who were then compared to a control group [120]. They found that RV infection induced a bacterial coinfection in 60% of the COPD (compared to 10% of the control group) with NTHi as the primary pathogen and in a follow-up study showed a peak of bacterial load at two weeks with bacteria present at least six weeks after infection [121]. This was in association with increased degradation of antimicrobial peptides. There have been several studies describing how RV infection compromises host immunity. RV infection has been shown to damage the tight junctions between epithelial cells allowing NTHi to move in between cells [122]. RV also inhibits macrophage interleukin 1 responses to NTHi and attenuates IL-8 responses via TLR-2 dependent degradation of IRAK-1 [123].

Influenza infection may also be complicated by secondary bacterial infection and this was particularly prevalent in the 1918-1919 Spanish flu outbreak in which it was a major cause of death (with* Pneumococcal *and* Haemophilus* infections being predominant) [124, 125]. The main mechanism leading to secondary bacterial infection in this circumstance is death of the virally infected epithelial cells, which destroys the structural integrity of the lower respiratory tract.

Viral infection can potentially have two important effects. It can lead to the initial infection of the lung with NTHi as what may occur in influenza infection. It may also be associated with exacerbations of infection in established lung disease as what occurs with rhinovirus.

## 8. Manipulating the Immune Response to NTHi

Generally NTHi is sensitive to standard antibiotics (although there is an increasing population of beta-lactamase-producing NTHi as well as strains beta-lactamase-nonproducing ampicillin-resistant (BLNAR) strains). However despite this, infection with NTHi is frequently recurrent or chronic. Therefore this bacterium may be able to establish a niche in the lower respiratory tract, where it is protected from the action of antibiotics. Therefore, manipulating the immune response to enhance host immunity is a potentially important method in the treatment of this bacterium.

Macrolide antibiotics in recent trials have been shown to reduce exacerbations of airways disease including COPD and bronchiectasis [126–128]. Macrolides have immunomodulatory as well as antibacterial effects.

There are other potential immune mediators that could be used to treat NTHi infection. These include stimulants of the immune system such as interferon gamma, which has been used to enhance immune response and decrease infections in the treatment of the inherited immune deficiency chronic granulomatous disease (CGD) [129, 130]. There are other reports of its use in the treatment of mycobacterial infection [131] and it can enhance monocyte killing of NTHi in vitro [101]. There are an increasing number of new antibodies that have recently become available to block specific cytokines such as anti-IL-4 and anti-IL-13. Such immune mediators could potentially have a role in the treatment of NTHi infection/inflammation, although their clinical use would depend upon a significantly improved understanding of the immune response to this bacterium. A recent study described that peroxisome proliferator-activated receptor-*γ* (PPAR-*γ*) activation by rosiglitazone was effective in preventing cigarette smoke-induced neutrophilia exacerbation following NTHi infection [132].

The most important treatment to improve outcome in the treatment of NTHi infection would be the development of an effective vaccine. The Hib vaccine, which induces a protective humoral immune response to the bacterial capsule, is highly effective [1, 133]. In contrast, there is not a standard, effective vaccine available for NTHi, which has a large number of different strains with extensive antigenic variation. There is extensive literature available on potential targets for vaccination and their use in animal models of upper airway disease. A few important studies are highlighted in this review. The outer membrane P2 is a potential candidate and has been shown to be associated with protective immunity in animals [134]. P2 does have significant variability and antibodies against it are strain specific and not protective against infection against different strains [88]. Another potential target for vaccination is protein D, which promotes adherence to epithelial cells [135]. In a murine model vaccination with protein D enhances clearance of NTHi from the ear and lung [136]; and a chinchilla model demonstrated benefit in ear infection [137]. It has been shown that patients with COPD have reduced IgG specific responses to protein D [138]. Antibodies to protein D have also been shown to correlate with protective immunity in humans [139]. There are a number of other candidates that have been tested for ability to induce protective antibodies and these include the adhesins HMW1/HMW2 and Hia [140], surface protein F [141], OMP26 [142], P4, and protein E. There are also studies that have assessed potential T cell vaccine responses. Gershon et al. used protein D and OMP26 to successfully stimulate memory T cell responses in children but not in adults [143]. OMP26 has been shown to activate T cells in another study [144]. There are a variety of other potential targets for vaccination that include the type IV pilus (Tfp) proteins of NTHi. The Tfp of NTHi has a number of important biological functions including its ability to facilitate adherence to epithelial cells. Carruthers el al. have recently defined biological roles of these proteins in the adherence to human airway cells and this work in combination with other studies in upper respiratory tract models has implications for a Tfp-derived vaccine [145].

There have been several oral vaccines that have been used to treat NTHi infections. Whole, killed NTHi has been used as a vaccine in patients with chronic bronchitis and studies reported a reduction in the frequency and severity of exacerbations although the data was suboptimal [146, 147]. A more recent study with a more refined bacterial substrate reported reduction in exacerbations [148], although subsequent follow-up studies have failed to confirm these findings. A conjugate vaccine (PHiD-CV11) of polysaccharides from 11 different* S. pneumoniae* serotypes conjugated to* H. influenzae* derived protein D was used in the prevention of acute otitis media [139]. The results showed that this conjugate vaccine allowed protection against pneumococcal otitis (58% for any episode) and also for* H. influenzae-*induced acute otitis media (35% reduction). This conjugate vaccine has subsequently been widely approved in a number of countries (now modified as PHiD-CV10). A subsequent vaccine combining 13 serotypes of pneumococcus conjugated to a diphtheria protein has been developed and this is the most commonly used conjugate vaccine for the pneumococcus; however, the recommendations do have some variations between different countries [94]. There have been limited subsequent studies assessing the role of PHiD-CV10 in the prevention of NTHi infection. There is a possible effect on nasopharyngeal NTHi colonization in children [94, 145]. A Finnish study in young children demonstrated that this vaccine was effective in reducing the need for antibiotics [149]. A Cochrane review assessed the efficacy of oral* H. influenzae* vaccines for the prevention of acute exacerbations of chronic bronchitis and COPD. Their conclusion was that these vaccines did not produce a significant decrease in the number or severity of exacerbations [150].

A recent study described the nasopharyngeal inoculation of 15 healthy human subjects with NTHi. This induced colonization and specific immunoglobulin responses. The model was safe and raises the possibility of using a human model for proof of concept studies of this exclusively human pathogen [151].

## 9. Conclusions and Future Perspective

Nontypeable* Haemophilus influenzae* is a bacterium that has had a relatively low profile amongst clinicians despite its prevalence and associated burden of disease. It is part of the normal microbiome of the nasopharynx but, when it moves from this location to the lower respiratory tract, it induces inflammation. The host immune response to this bacterium is still not well defined. The development of more effective therapies for treatment of disease induced by NTHi is dependent upon improving the understanding of the host immune response.

Defining the immune response to a bacterium that is present chronically in the human host as a commensal but also is able to cause inflammation and significant disease is inherently challenging. There are some priority areas, which are important in advancing the understanding of the immune response to this bacterium summarized as follows.

*Potential Priority Areas for Future Investigation*

- Establishment of chronic models of infection and relevant knockouts.
- More detailed analysis of human lung responses to NTHi.
- Further defining the effects of smoking and viral coinfection with the burden of clinical disease.

NTHi is an exclusively human pathogen, which is particularly associated with chronic disease; and the development of representative animal models of this infection has been difficult to achieve. There is a need for chronic animal models of NTHi infection/inflammation to more closely represent the clinical situation. It may be hard to ascertain whether measured adaptive immune responses are a primary cause or effect of chronic/recurrent NTHi infection; the use of relevant knockout models will clarify such issues. As NTHi primarily causes disease in the respiratory tract/lung, studies of human lung tissue need to be prioritized. The majority of the studies that have been completed so far have used BAL macrophages. The study of other cell types and of surgical lung tissue is likely to yield important insights. Finally the study of smoking-related effects and viral coinfection is potentially of great relevance as these are the factors most clearly associated.
